# Health Seeking Behaviours among Caretakers of Children with Nodding Syndrome in Pader District - Northern Uganda: A Mixed Methods Study

**DOI:** 10.1371/journal.pone.0159549

**Published:** 2016-07-29

**Authors:** Pamela Atim, Emmanuel Ochola, Stephen Ssendagire, Elizeus Rutebemberwa

**Affiliations:** 1 Department of Health Policy, Planning and Management, Makerere University School of Public Health, Kampala, Uganda; 2 Department of Public Health, Gulu University Faculty of Medicine, Gulu, Uganda; 3 Department of HIV, Research and Documentation, St. Mary's Hospital Lacor, Gulu, Uganda; Academic Medical Centre, NETHERLANDS

## Abstract

**Background:**

Nodding syndrome is a neurological disorder which had affected about 3000 children with over 170 deaths in northern Uganda by 2012. With limited data on health seeking, the study aimed to assess the health seeking behavior and associated factors among caretakers of children with nodding syndrome in Pader district.

**Methods:**

A mixed methods cross sectional study was conducted in July 2013 among 249 caretakers of children with nodding syndrome in three sub-counties of Pader. Respondents were consecutively interviewed using semi-structured questionnaires. Eleven key informants were additionally interviewed. We determined the associations of various factors with health care seeking and obtained adjusted odds ratios and 95% confidence intervals using logistic regression model. Quantitative data was analysed using Stata version 12 while qualitative data was analysed manually and quotes reported.

**Results:**

Most caretakers, 78.3% (195/249) sought care first from a health facility, 12.9% (32/249) visited traditional healers and 8.8% (22/249) self-medicated. Of those who sought care from a health facility, 50% sought care after a month. Factors associated with improved care seeking included: Time taken to reach care 1–3 hours; adjusted odds ratio = 6.4 (95% CI = 2.96–14.03), time spent in care above five years; adjusted odds ratio = 12.0 (95% CI: 1.24–117.73) and changed care seeking place; adjusted odds ratio = 17.2 (95% CI: 3.64–81.67).

**Conclusion/ Recommendation:**

Caretakers sought care from multiple places. One in five caretakers still sought care outside a formal health facility. Many respondents who sought care first from health facilities went late, at least one month after symptoms onset. Factors associated with health seeking included distance, duration in treatment and not having changing care provider. There is need for massive sensitization of community to enhance prompt care seeking. More research is needed to elucidate the cause, thus finding the treatment for nodding syndrome, to prevent "wandering in hope".

## Introduction

Nodding syndrome is an emerging progressive neurologic disorder that affects mainly children and adolescents aged 5 to 15 years. It is characterized by involuntary head nods which are precipitated at the sight of food and cold weather. Other symptoms include multiple convulsions, mental retardation, physical deterioration and disability, malnutrition and stunted growth [1,2,3,4].

The possible cause of nodding syndrome has remained unclear, although more than 90% of affected children were infested with *Onchocerca volvulus*, a filarial worm, which is transmitted by the black fly, *Simulium species*, breeding near fast flowing rivers [3,5]. Nodding syndrome has only been described in sub-Saharan countries, including Tanzania in the 1960s, South Sudan in the late 1990s and Uganda from 2003 [4]. Recent attacks are localized and predominant in populations that were previously displaced [6]. In Uganda, by 2012, the incidence of nodding syndrome was 3000 children, with an incidence of 7 in 1000 children being affected in the northern districts of Pader, Kitgum and Lamwo, 170 of which were fatal. The highest burden of the illness was reported in Pader and Kitgum districts with about 1,488 and 1,278 cases respectively; and 66 and 98 deaths respectively [2,7,8].

There is no known definitive treatment for nodding syndrome. The Ugandan ministry of health responded by a multi-sectoral management approach. Three treatment centres for nodding syndrome were set up in the most affected sub-counties, where symptomatic management was provided, with anticonvulsant therapy, wound care, nutritional and psychosocial support, among others. A treatment protocol was developed and health care workers oriented in symptomatic nodding syndrome care [2,9,10].

Several studies were conducted since 2009, detailing magnitude, presentation outcomes and interventions for nodding syndrome [11,8,6,12,9,13]. One qualitative study [14] determined a multiple source treatment seeking, from biomedical health facilities, traditional medicine, and spiritual healers, but none of the studies has quantified the health seeking patterns among caregivers for nodding syndrome. Elsewhere in India, care seeking for epilepsy, a related illness was shown to be simultaneous; with 81.6 percent of the population opting for allopathic medicine, while others sought homeopathic care and traditional medicine [15]. In this study, we examined the patterns of health seeking behaviours of caretakers of children with nodding syndrome, as well as the factors associated with those patterns.

## Materials and Methods

In July 2013, we conducted a mixed methods cross sectional study in three sub-counties of Pader district to collect data on health seeking behavoiurs of caretakers with nodding syndrome children and its associated factors. Pader district was chosen because it had the highest number of children with nodding syndrome, 1488 of the 3000 cases in northern Uganda [2].

### Participants and sampling

A statistically determined sample of 249 households which had a person suffering from nodding syndrome was obtained in the three most affected sub-counties by sampling proportionate to number of nodding syndrome patients. From these households we interviewed adult caretakers of the children with nodding syndrome, since we believe they are in position to provide information about the chronology of health seeking from the onset of nodding syndrome symptoms. Respondents were selected consecutively until the required sample was achieved in each sub-county.

An additional 11 key informants were selected purposively, including three health care workers, two drug shop dispensers, five community leaders and one religious leader. All respondents were above 18 years of age.

### Data collection and procedures

Trained research assistants obtained informed consent before administering a pretested questionnaire (S1 Questionnaire) to the caretakers of children with nodding syndrome, collecting information on nodding syndrome in the child, its onset and health seeking course, as well as perceived cause.

Prior to data collection, the study team worked with the health assistant attached to the treatment centres in each sub-county, to mobilise the caretakers for the interviews, which took place at the community meeting venues. Such a venue is commonly in a large open space, with trees or houses. Privacy was observed during interview, by conducting it in a house or under an isolated tree in the compound.

Key informants were sought in their offices and interviewed to provide qualitative corroborating information regarding nodding syndrome health seeking in their communities. Qualitative findings were integrated with the quantitative findings at results and interpretation stage. All interviews lasted about 20–30 minutes.

### Data analysis

Quantitative data was entered in Epidata and exported to Stata version 12 for analysis. We displayed descriptive statistics for categorical variables into frequencies, percentages and proportions. Open ended answers were grouped and frequencies provided. Logistic regression was used to determine associations between predictors and health seeking behavior. Unadjusted and adjusted odds ratios and 95% confidence intervals were reported. Qualitative data from key informants was analysed manually into themes and quotes were reported based on repetitive and unique issues.

### Human subject

We obtained ethical clearance and approval from Makerere university school of Public Health research and ethics committee before conducting this study. Permission was also obtained from Pader district health office, and caretakers of nodding children provided written informed consent. Participants were interviewed in privacy and unique identifiers were used at data entry for confidentiality.

## Results

We interviewed 249 caretakers of children suffering from nodding syndrome and 11 key informants. Majority of the respondents were women, 77.5% (193/249) and Catholics72.7% (181/249). Almost all the respondents were Acholi, 99.2% (247/249) and nearly half of them had informal education, 48.6% (121/249) while 49.0% (122/249) had attained primary education. Majority of the caretakers were peasants farmers, 98.4% (245/249) and at least 30% (73/249) had more than one child with nodding (Table 1).

**Table 1:** Socio-demographic characteristics of households.

Variable	Frequencies (N = 249)	Percentages (%)
**Sex**		
Male	56	22.5
Female	193	77.5
**Marital status**		
Single	38	4.8
Married	170	68.3
Divorced/separated	21	8.4
Widowed	46	18.5
**Education**		
None/Informal	121	48.6
Primary	122	49.0
Secondary	04	1.6
Tertiary	02	0.8
**Religion**		
Catholic	181	72.7
Protestant	42	16.9
Pentecostal	24	9.6
Traditionalist	02	0.8
**Tribe**		
Acholi	247	99.2
Lango	02	0.8
**Occupation**		
Peasant	245	98.4
Civil servant	01	0.4
Business	03	1.2
**No. nodding children in household**		
One	176	70.7
Two	55	22.1
Three	9	3.6
More than three	9	3.6

### Health seeking behavior of caretakers of children with nodding syndrome

**Fig 1:**
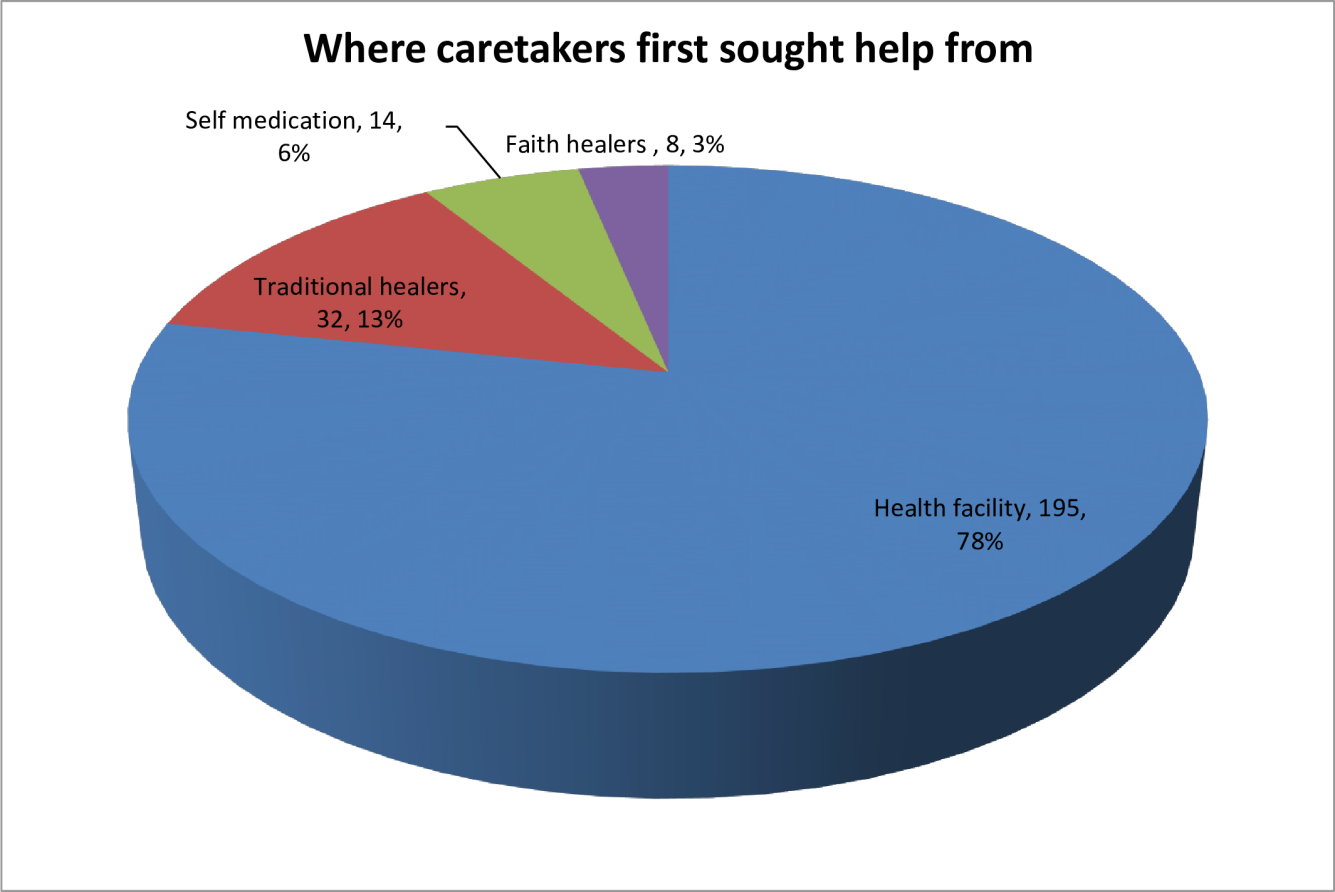
Places for health seeking for nodding syndrome.

Caretakers of nodding children sought care from multiple places. Majority sought care first from a health facility, 78.3% (195/249), about 12.9% (32/249) went to a traditional healer, and 3.2% (8/249) sought first care from faith based healers (Fig 1). Of those going first to a health facility, 50% sought care at least one month after onset of symptoms.

### Reasons for choice of particular treatment options

The reasons for seeking care from different places varied. Those who sought care at health facility, the commonest reasons were "hope for improvement or cure for their children (50%), or that they knew the system works, or they knew of no other place to seek help from, or that the presentation warranted health seeking”. Some eight percent went because they had seen others improve, while some seven percent went because they were advised by others. Of those who visited the traditional healers, about 32% said they were advised by others, while about 26% hoped they would get treatment or even cure. Uniquely some 18% of these went because they thought it was epilepsy, while 14% went because they thought it was demonic possessions or evil spirits. The main reasons for going to church or faith based healers were because they thought it was demonic possession/spiritual in 50%, or that they hoped for cure since God is the healer. Half of those who had self-treatment or went to drug shops did so because they hoped for cure since they thought it was simple presentation; another 20% said they were advised, while close to 15% resorted to self-medication due to lack of treatment for nodding syndrome at health facilities at that time.

### Alternative care seeking following the initial health seeking

Of the 249 respondents, majority 77.1% (192/249) changed care seeking place after the first care sought. In fact, most of them went to a health facility (90.6%, 174/192). Actually they also moved from a lower health centre to another, seeking for better treatment. However, the other 9.4% (18/192) still went to traditional healers, faith healers, or drug shops.

### Factors associated with health seeking

At a multivariable analysis, factors associated with health seeking were time taken to reach place of care, time in care and whether respondent changed care seeking place or not. Caretakers who took 1–3 hours and more than 3 hours as compared to those taking less than an hour to reach the treatment site were about 6 and 5 times respectively more likely to have sought care in a health facility, adjusted odds ratio (aOR 6.4, 95% CI:3.0,14.0), more than 3 hours (aOR 5.3, 95% CI: 1.2, 23.4). Compared to those who have lasted one year in care, respondents who have spent more than five years in one care option were 12 times more likely to have gone to a health facility (aOR 12, 95% CI: 1.2, 117.7). Those who did not change treatment seeking place were 17 times more likely to have gone to a health facility compared to those who changed treatment (aOR 17.2, 95% CI: 3.6, 81.7). Socio-demographic determinants like marital status, educational level of respondent, religion, convulsion, number of children in the household and occupation were not associated with health seeking behavior (Table 2).

**Table 2:** Multivariable logistic regression analysis associated with health care seeking.

Variable	Freq, N = 315*	% Health care seeking	aOR (95%CI)	P-value
**Time to facility**				
Less than 1hour	113	22.9	1	
1hr-3hours	184	50.8	6.4 (3.0,14.0)	<0.001
More than 3 hours	18	4.8	5.3 (1.2,23.4)	0.028
**Time in care**				
Less than 1 year	105	19.7	1	
1–5 years	164	44.4	1.9 (0.9,4.0)	<0.001
More than 5 years	46	14.3	12.1 (1.2,- 117.7)	0.032
**Changed care option**				
Yes	191	40.0	1	
No	124	38.4	17.2 (3.6,81.7)	<0.001

*For logistic regression to determine associated factors, the analysis was based on the total 315 children of the 249 parents, since from Table 1, 73 households had more than one child with nodding syndrome.

Qualitative data also documented references to high levels of health seeking from many providers such as traditional healers, formal health facility, faith healers and drug shops/ clinics. For instance many people sought care either from health facility or traditional healers but others first did self-medication. The facility where treatment/care was sought from depended on the perceived cause of the disease. If caretakers of nodding patients believed that the cause of nodding was due to ghost or evil spirits, they tended to go to traditional healers. One Local Council leader from Laguti Sub-county said:

"I think it's a disease that doesn't want children to eat. May be it is a kind of spirit, or maybe since we had war for a long time, maybe those killed innocently say, "Since I was killed without food-in hunger, other people should also die like I died."

Interviews with drug shop owners further confirmed that many caretakers of children with nodding syndrome shift getting care from one place to the other.

*"In the past, before the establishment of treatment centres, many people would come to buy drugs from drug shops. Medicines San Frontiers (MSF), a non-governmental organisation was also helping treat nodding patients at Atanga Health Centre (HC) III. Nowdays, people who have money take their children to Gulu or Kitgum hospitals (*neighbouring districts) *while others go to Atanga HC III. Others were using local traditional herbs because they thought their children had epilepsy while others go to consult witchdoctors thinking the disease was caused by evil spirits*."

Many health care workers in health centres agreed that before establishment of treatment centres for nodding syndrome, many caretakers would wander from one health unit to another. But even with the establishments of treatment centers many caretakers still moved from one place to another in hope of seeking cure for their children. Some people go to a higher level health centre (Health centre IV) or to a hospital, with the hope of getting better treatment or definitive diagnoses. One health worker at a health centre III said,

"Many parents are usually disappointed that their children do not improve very significantly and quickly. So they wander from one health unit to another, or go to district and referral hospitals in Gulu and Kitgum. But somehow this has improved after the institution of treatment centres for nodding syndrome patients.

## Discussion

Our findings in Pader district in northern Uganda have quantified that eight in ten caretakers of children with nodding syndrome sought care first from health facilities while one in ten sought care first from traditional healers and five percent provided self-medication for their children either by buying drugs from drug shops or giving them herbs. Although most caretakers of children with nodding syndrome seek care first from a health facility, many go late. Furthermore, interviews with key informants revealed that caretakers tend to seek care concurrently from different places. This is consistent with a study from northern Uganda among nodding syndrome caretakers [14] and a Tanzanian study among people with epilepsy, which found that almost all people with these chronic illnesses demonstrated a pluralistic care-seeking behavior that included use of traditional medicine and prayers beside modern medicine [16]. The pluralistic care seeking is a common practice among Ugandans [17,18] for chronic diseases, which has implications on the management of such patients in terms of adherence to care schedules, generating the need for professionals to develop strategies for improving client consistency in biomedical care.

Our study also found that many people still seek care outside the recommended places such as consulting traditional healers or witchdoctors, or soliciting unprescribed medicines. This has implication on health education and community awareness. The use of self-medication or basic drug shops where a person just asks for particular drugs without prescription is not encouraged, however, this was found in 5% of our respondents. Findings from western Uganda revealed that up to 43% of respondents sought care for malaria in drug shops [19].The variance from our study is possibly because of a more definitive manifestation of malaria.

Despite a high proportion of caretakers of children with nodding syndrome seeking care first from a health facility, only 50% sought health care within one month when symptoms appeared. Many of the caretakers took up to one year without seeking care. This possibly rhymes with the vagueness of the symptoms of nodding syndrome. This finding is in line with findings that the rate of health care seeking is low for symptoms which are chronic in nature [20]. What is interesting is that when these caretakers take action, they are more likely to report to a health facility. Slow and/or late seeking of care from a formal health facility has big implications for the management of nodding syndrome. Late presentation for health care can impair the extent of effective management; hence this might suggest the need for creating awareness among the people by the health workers.

Perceived knowledge of cause of disease has been found to affect health seeking behavior [16] however the limited knowledge about the cause of nodding syndrome could not allow an exhaustive examination of this factor. Also, we had got a significant association between convulsion (clinical manifestation of nodding syndrome) and health seeking behavior at bivariable analysis but there was no association at multivariable analysis. This finding is in disagreement with findings by De Savigny et al that caregivers would seek help from a health facility irrespective of whether the children had convulsions or not in malaria [21]. It is best understood from the context of how nodding syndrome presents. Interviews with caretakers of these children showed that convulsion is a later feature of disease presentation and usually manifest when the disease has progressed in a child.

We found that respondents who took more than one hour as compared to those taking less than an hour to reach the treatment site were at least five times more likely to seek care in a health facility. Time taken to reach facility directly relates to distance to facilities. About one to three hours represented time caretaker would take to reach the health facility from their home when footing, possibly to reach a facility within 5 kilometers as recommended by the Ministry of health. Distance to a health facility is one of the delays to seeking care from a health facility. This finding was in agreement with other studies [21]. Could it be that those who live near the facilities see gaps in management of nodding syndrome in those facilities? Compared to those who have lasted one year in care, respondents who have spent over five years in care were 12 times more likely to have gone to a health facility. Those who did not change treatment seeking were 17 times more likely to have gone to a health facility compared to those who changed treatment. So after all, health facilities, far or near, remained an important source of care seeking.

Among the reasons cited as drivers for seeking care at health facilities was hope of getting cure for the children. Interview with caretakers of children with nodding syndrome as well as health workers and community leaders revealed that most caretakers confessed that with the medicine got from the health units, their children greatly improved and some started eating. This can further be supported by a huge likelihood of changing treatment if you did not start from a health facility. Our study found that compared to those who changed the nature of treatment sought; those who did not change treatment were 20 times more likely to have gone to a health facility. This implies that in the end, everybody switches to a formal health care system. The implication of many seeking care from a health facility is better management of the cases. The finding agrees to what Mitchel, Kornfeld et al found about perceived treatment got from the health facility, that it controls seizures but does not chase away evil spirits [22], leading to partial health seeking.

Many caretakers gave reasons of hope for cure or belief in competence of the sought system for the health of their children. This is in line with reasons another study gave on convenience, or trust in the treatment care system. According to Mitchell et al., caretakers reported that the only positive change they saw in their children was as a result of the antiepileptic drugs given from the health facilities [20]. The health system must thus prove itself as delivering, even as the caretakers wander from one facility to another in hope of finding a definitive solution.

### Study limitations

The vague nature of initial presentation of nodding syndrome, coupled with the lack of a control group could potentially impair the specificity of the responses on health seeking behaviour to nodding syndrome. To counter this, our study only enrolled caretakers of children who were already in care for nodding syndrome, verified by medical forms and the list from the health facilities. Since caretakers were expected to narrate the history of health seeking, some of which occurred a long time before, the risk of recall bias in our study was reduced by probing.

## Conclusion

Our study found that 80 percent of caretakers of children suffering from nodding syndrome seek care first from health facility, but a significant one in ten still seek care with traditional healers, and five in 20 go for self-treatment. Of those who seek care first from a health facility, only half of the caretakers sought immediate health care for nodding syndrome within a month, some of the caretakers took up to one year without seeking care. Belief in the competence of the sought health system was a big driver in the nature of health sought. Apart from that, the strange nature of the disease also made many clients to wander from one place to another seeking for definitive diagnosis or cure.

There is need for massive sensitization of community to enhance prompt care seeking. Ministry of health and health workers should deliver optimal health services to manage nodding syndrome patients. More research needs is needed to elucidate the cause, thus finding the treatment for nodding syndrome, to prevent "wandering in hope".

## Supporting Information

S1 Questionnaire Questionnaire and interview guide. (DOC)
