# Supplementary material for: Health Seeking Behaviours among Caretakers of Children with Nodding Syndrome in Pader District - Northern Uganda: A Mixed Methods Study
Source: PLoS One. 2016 Jul 29;11(7):e0159549. doi: 10.1371/journal.pone.0159549 (PMC4966934; doi:10.1371/journal.pone.0159549)
Supplement: S1 Questionnaire — (DOC) [file pone.0159549.s001.doc]

Appendix V: Questionnaire

| Household ID |  |  |  |  |  |  |  |
| --- | --- | --- | --- | --- | --- | --- | --- |
|  |  |  |  |  |  |  |  |
| Date of Interview |  |  |  |  |  |  |  |
|  |  |  |  |  |  |  |  |
| District |  |  |  |  |  |  |  |
|  |  |  |  |  |  |  |  |
| Sub-county |  |  |  |  |  |  |  |
|  |  |  |  |  |  |  |  |
| Parish |  |  |  |  |  |  |  |
|  |  |  |  |  |  |  |  |
| Village |  |  |  |  |  |  |  |

Name of Interviewer: ………………………… ……………….. Phone. No:

|  | **PLEASE INDICATE MOST APPROPRIATE RESPONSE Response** | |
| --- | --- | --- |
| 1 | Age of the child (in completed years) |  |
| 2 | Sex of the child   1. Female 2. Male |  |
| 3 | What is the age in completed years of respondent? |  |
| 4 | Sex of the respondents  1. Female  2. Male |  |
| 5 | Tribe of respondent   1. Acholi 2. Langi 3. Itesot 4. Madi 5. Lugbara 6. Others (Specify)…………………………………………………………………………. |  |
| 6 | Religion   1. Catholic 2. Protestant 3. Muslim 4. Others (specify)………………………………………………………………………….. |  |
| 7 | Educational level of respondent   1. Graduate 2. Diploma 3. Certificate 4. Completed A level 5. Completed P.7 6. Never went to school 7. Others (specify)……………………………………………………………………….. |  |
| 8 | What is the primary occupation of respondent?   1. Peasant farmer 2. Civil servant 3. Businessman/ woman 4. Politician 5. Causal worker 6. Unemployed 7. Others (specify)……………………………………………………………………….. |  |
| 9 | What is the marital status of the respondent?   1. Married and monogamous 2. Married and polygamous 3. Single 4. Separated 5. Divorced 6. Widow 7. Widower 8. Others (specify)………………………………………………………………….. |  |
|  | **HEALTH SEEKING BEHAVIOUR (Child I)** |  |
| 10 | How many children do you have? | ------------- |
| 11 | How many of them are suffering /suffered from nodding syndrome?   1. One 2. Two 3. Three 4. More than three If more than one please fill particulars for child II |  |
| 12 | For how long has the child been with the disease?   1. Less than six months 2. More than six months 3. One year 4. Others (specify)………………………………………………………………………. |  |
| 13 | What do you think causes nodding syndrome?   1. Black flies 2. War 3. Displacement in the camps 4. Don’t know 5. Others (specify)……………………………………………………………………… |  |
| 14 | When this child had nodding, what signs and symptoms did you observe? Circle all that apply   1. Stopped eating 2. Head nodding only 3. Head nodding and convulsions 4. Others (specify)………………………………………………………………………. |  |
| 15 | When did the child start showing signs and symptoms? Please indicate at least month and year |  |
| 16 | Did you seek any form of care for this child?   1. Yes 2. No If Yes, skip question 17 and go to Qn. 18 |  |
| 17 | If No, why? ………………………………………………………………………………………………………… |  |
| 18 | If Yes, how soon did you seek care?   1. Immediately 2. Within two weeks 3. Within one month 4. More than a month 5. Others (specify)……………………………………………………………………………. |  |
| 19 | Where first did you seek care from?   1. Health facility 2. Traditional healer 3. Religious/Faith healers 4. Self- treatment 5. Others (specify)…………………………………………………… If Health facility go to Qn. 20, otherwise proceed to Qn. 21 |  |
| 20 | Please indicate type and level of Health facility   1. Government health facility 2. Health centre IV 3. Health centre III 4. Health centre II 5. Private health facility |  |
| 21 | What is the estimated distance from your home to this place indicated in qn. 19 above?   1. Less than 5 Kms 2. More than 5 Kms 3. Others (specify)………………………………………………………. |  |
| 22 | How long does it take you to reach the place of care mentioned in qn. 19 above?   1. Less than 30 minutes 2. About 1 hour 3. More than 1 hour |  |
| 23 | For how long has the child been on that care?   1. About three months 2. Six months 3. 1 year 4. More than 2 years |  |
| 24 | Why did you opt for this type of care?...........................................................................................  ……………………………………………………………………………………………………  ………………………………………………………………………………………………….. |  |
| 25 | Who advised you on that care?   1. Self 2. Husband 3. Sister 4. Grand-mother 5. Mother-in law 6. Others (specify)……………………………………………………………………………. |  |
| 26 | Did you have to change treatment from the first mentioned in qn. 19 to other places?   1. Yes 2. No If Yes, proceed to qn. 27 |  |
| 27 | If yes, where? ………………………………………………………………………………… |  |
| 28 | Are you still getting treatment from there?   1. Yes 2. No |  |
| 29 | Why did you change to getting treatment from there?   1. Free services 2. Less cost 3. Short distance 4. Availability of drugs 5. Less waiting time 6. Others (specify)…………………………………………………………………………. |  |
| 30 | Where else did you seek help/ treatment from for this child? Indicate all options  …………………………………………………………………………………………………...  …………………………………………………………………………………………………... |  |
| 31 | Give reason for each place of help in 30 above  …………………………………………………………………………………………………..  ………………………………………………………………………………………………….. |  |
|  | **Child II** |  |
| 31 | Age of the child (in completed years) |  |
| 32 | Sex of the child   1. Female 2. Male |  |
|  | **HEALTH SEEKING BEHAVIOUR FOR CHILD II** |  |
| 33 | For how long has the child been with the disease?   1. Less than six months 2. More than six months 3. One year 4. Others (specify)………………………………………………………………. |  |
| 34 | When the child had nodding syndrome, what signs and symptoms did you observe?   1. Stopped eating 2. Head nodding only 3. Head nodding and convulsions 4. Others (specify)……………………………………………………………….. |  |
| 35 | When did the child start showing signs and symptoms? Please indicate at least month and year |  |
| 36 | Did you seek any form of care for this child?   1. Yes 2. No If Yes, skip question 36 and go to Qn. 37 |  |
| 37 | If No, why?  …………………………………………………………………………………………………… |  |
| 38 | If Yes, how soon did you seek care?   1. Immediately 2. Within two weeks 3. Within one month 4. More than a month 5. Others (specify)…………………………………………………………………………… |  |
| 39 | Where first did you seek care from?   1. Health facility 2. Traditional healer 3. Religious/Faith healers 4. Self-treatment 5. Others (specify)…………………………………………………………………. If at health facility go to Qn. 39, otherwise proceed to qn.40 |  |
| 40 | Please indicate type and level of health facility   1. Government health facility 2. Health centre IV 3. Health centre III 4. Health centre II 5. Private non-for profit health facility. Indicate level |  |
| 41 | What is the estimated distance from your home to this place indicated in question 19 above?   1. Less than 5 Kms 2. More than 5 Kms 3. Others (specify)………………………….. …………………………………………. |  |
| 42 | How long did it take you to reach that place?   1. Less than 30 minutes 2. About 1 hour 3. More than 2 hours 4. Others (specify)……………………………………………………………………….. |  |
| 43 | For how long has the child been on that care?   1. About three months 2. Six months 3. 1 year 4. Others (specify)………………………………………………………………………… |  |
| 44 | Why did you opt for this type of treatment/care?  ……………………………………………………………………………………………………  ……………………………………………………………………………………………………  …………………………………………………………………………………………………… |  |
| 45 | Who advised you on that care/treatment?   1. Self 2. Husband 3. Sister 4. Mother-in-law 5. Grand mother 6. Others (specify)…………………………………………………………………………………….. |  |
| 46 | Did you have change care/treatment from the first one mentioned in qn. 38 to other places?   1. Yes 2. No If Yes, proceed to qn. 45 |  |
| 47 | If Yes, where?  ………………………………………………………………………………………………………….. |  |
| 49 | Are you still getting treatment from there?   1. Yes 2. No |  |
| 50 | Why did you change to getting treatment from there?   1. Free services 2. Less cost 3. Short distance 4. Availability of drugs 5. Others (specify)…………………………………………………………………………………. |  |
| 51 | Where else did you seek help/treatment from for this child?  ………………………………………………………………………………………………….. |  |
| 52 | Why?............................................................................................................................................. |  |

**THANK YOU FOR YOUR TIME!!!!!!**

## Appendix vii: Key Informant Interview (KII) Guide

**Topic of the study:** Patterns of health seeking behviour and its associated factors among caretakers of children with Nodding Syndrome in Pader district

**Part A**: Key Informant

Name……………………………………………Designation…………....................................

Date of interview: ……………………………………………………………………………..

Start time: ……………………………………..End time: …………………………………….

Name of Moderator: ……………………………………………………………………………

1. Briefly tell me about Nodding Syndrome. Probe for:

- when first case was reported
- Prevalence in district or health centre
- What causes it
- Any differences in causes across districts like Kitgum and Lamwo?
- What are the signs and symptoms
- How it is spread

1. When a child in this village falls sick with nodding syndrome, where do caretakers most seek treatment from?
2. In your opinion, what factors influence the decision to seek care from particular places mentioned above? Probe for:

- Reasons for visits
- Who advises them
- Most visited places on first attempt

1. What challenges are there in utilizing available services for Nodding syndrome in district?
2. What recommendations do you give to improve early identification and health seeking for this condition?
